# Boost the Crystal Installation and Magnetic Features of Cobalt Ferrite/M-Type Strontium Ferrite Nanocomposites Double Substituted by La^3+^ and Sm^3+^ Ions (2CoFe_2_O_4_/SrFe_12−2x_Sm_*x*_La_*x*_O_19_)

**DOI:** 10.3390/ma14247820

**Published:** 2021-12-17

**Authors:** Mahmoud M. Hessien, Ali Omar Turky, Abdullah K. Alanazi, Mohammed Alsawat, Mohamed H. H. Mahmoud, Nader El-Bagoury, Mohamed M. Rashad

**Affiliations:** 1 Advanced Materials and Applied Metallurgy Group, Department of Chemistry, College of Science, Taif University, P.O. Box 11099, Taif 21974, Saudi Arabia; aalanaz4@hotmail.com (A.K.A.); mosawat@tu.edu.sa (M.A.); mheshamm@gmail.com (M.H.H.M.); 2 Central Metallurgical Research and Development Institute (CMRDI), P.O. Box 87, Helwan 11421, Egypt; naderelbagoury@yahoo.com (N.E.-B.); rashad133@yahoo.com (M.M.R.); 3 Academy of Scientific Research and Technology, Cairo 11516, Egypt

**Keywords:** magnetic composite, Sr-Hexaferrite, cobalt ferrite, synthesis, morphology, magnetic property, annealing temperature

## Abstract

Spinel cobalt ferrite/hexagonal strontium hexaferrite (2CoFe_2_O_4_/SrFe_12−2x_Sm*_x_*La*_x_*O_19_; *x* = 0.2, 0.5, 1.0, 1.5) nanocomposites were fabricated using the tartaric acid precursor pathway, and the effects of La^3+^–Sm^3+^ double substitution on the formation, structure, and magnetic properties of CoFe_2_O_4_/SrFe_12−2x_Sm*_x_*La*_x_*O_19_ nanocomposite at different annealing temperatures were assayed through X-ray diffraction, scanning electron microscopy, and vibrating sample magnetometry. A pure 2CoFe_2_O_4_/SrFe_12_O_19_ nanocomposite was obtained from the tartrate precursor complex annealed at 1100 °C for 2 h. The substitution of Fe^3+^ ion by Sm^3^–^+^La^3+^ions promoted the formation of pure 2CoFe_2_O_4_/SrFe_12_O_19_ nanocomposite at 1100 °C. The positions and intensities of the strongest peaks of hexagonal ferrite changed after Sm^3+^–La^3+^ substitution at ≤1100 °C. In addition, samples with an Sm^3+^–La^3+^ ratio of ≥1.0 annealed at 1200 °C for 2 h showed diffraction peaks for lanthanum cobalt oxide (La_3_Co_3_O_8_; dominant phase) and samarium ferrite (SmFeO_3_). The crystallite size range at all constituent phases was in the nanocrystalline range, from 39.4 nm to 122.4 nm. The average crystallite size of SrFe_12_O_19_ phase increased with the number of Sm^3+^–La^3+^ substitutions, whereas that of CoFe_2_O_4_ phase decreased with an *x* of up to 0.5. La–Sm co-doped ion substitution increased the saturation magnetization (*Ms*) value and the subrogated ratio to 0.2, and the *Ms* value decreased with the increasing number of double substitutions. A high saturation magnetization value (*Ms* = 69.6 emu/g) was obtained using a La^3+^–Sm^3+^ co-doped ratio of 0.2 at 1200 for 2 h, and a high coercive force value (*Hc* = 1192.0 Oe) was acquired using the same ratio at 1000 °C.

## 1. Introduction

The consolidation of spinel soft and hard ferrites in nanocomposites can considerably change the magnetic characteristics related to interfacial exchange coupling. Composite magnets comprising soft phases with high saturation magnetization, and hard phases with high coercivity, can be merged as permanent magnets. Nanocomposite magnets offer wide-band absorption, with a megahertz (spinel ferrites MFe_2_O_4_, where M = divalent ion, such as Cu, Zn, Cd, Co, Ni, Mn, or mixture of two divalent ions) to gigahertz (hard ferrites) range. This feature can be useful for minimizing the radar cross-section of the target and problems due to electromagnetic interference over wireless communications [1,2,3]. Hard hexagonal ferrites are classified as M-type hexaferrites, including MFe_12_O_19_, Y-type ferrites (M_2_Me_2_Fe_12_O_22_), W-type ferrites (MMe_2_Fe_16_O_27_), X-type ferrites (M_2_Me_2_Fe_28_O_46_), U-type ferrites (M_4_Me_2_Fe_36_O_60_), and Z-type ferrite (M_3_Me_2_Fe_24_O_41_), where M is Ba, Sr, or Me is a small ionic radius such as Zn, Co, Ni [4]. Strontium hexaferrite (SrFe_12_O_19_) M-type ferrite is the most well-known hard ferrite, owing to its merits, such as low cost, high saturation magnetization, high magnetization, high coercive force, excellent corrosion resistance, eminent chemical stability, and high Curie temperature (470 °C). It has a wide application range, in the fields of automotives, home appliances, electronics, lighting, biomedical and diagnostic applications, and oil and energy [5]. In addition, the commercial applications of M-type hexaferrite for high-density recording media depend on the ferrite type. The production of major recording media and permanent magnets require inexpensive ferrites with good chemical stability and high resistance. For recording media such as video type, CVD, DVD, and USB, high coercivities complicate re-recording. However, high coercivity prevents the corruption of information in credit and identification cards exposed to stray magnetic fields [6]. The exchange coupling of CoFe_2_O_4_ as a spinel ferrite and SrFe_12_O_19_ as a hard ferrite composite has been scrutinized [7]. Dhabekar and Kant [8] synthesized a cobalt ferrite/strontium hexaferrite nanocomposite in a ratio of 1:1, with a facile co-precipitation strategy. The change in the dielectric properties of the elaborated composite was investigated at different temperatures, ranging from 100 °C to 400 °C. Using a similar technique, Dhabekar and Kant [9] fabricated a CoFe_2_O_4_/SrFe_12_O_19_ nanocomposite (1:2) with a saturation magnetization of 47.02 emu/g. However, Pan et al. [10] processed CoFe_2_O_4_/SrFe_12_O_19_ nanocomposites based on an electrospinning pathway at different Co^2+^/Sr^2+^ ratios. The maximum saturation magnetization of the nanocomposite was 62.8 emu/g. Panchal and Jotania [11] prepared a CoFe_2_O_4_-SrFe_12_O_19_ composite using the Self-propagating high-temperature synthesis SHS approach. The magnetic features and the particle size were monitored on the basis of the variations in the content of spinel ferrite in the composite with hexagonal strontium ferrite. Petrecca et al. [12] attempted to synthesize SrFe_12_O_19_/Zn_1-*x*_Fe_3_O_4_ nanocomposite by milling the materials in a weight ratio of 90:10 in mortar and then annealed at temperatures of 500–1100 °C for 2 h. Their aim was to expand the application range of the nanocomposite for moderate energy product applications involving the automotive and energy industries. Meanwhile, Algarou et al. [13] developed SrFe_12_O_19_ and Mg_0.5_Cd_0.5_Dy_0.03_Fe_1.97_O_4_ nanocomposites for antifungal applications. The electrochemical performance of SrFe_12_O_19_/CoFe_2_O_4_ nanocomposite synthesized using the hydrothermal pathway was investigated. The specific capacitance was increased from 133 F g^−1^ in pure Sr-ferrite to 634 F g^−1^ at a Co/Sr ratio of 0.5 [14]. The magnetic properties of M-type hexaferrite was enhanced by the partial replacement of Sr or Fe or both. For example, the possibility of substituting Sr by La creates an increment of the magneto-crystalline anisotropy. Therefore, the impact of other rare earth elements, such as Nd and Sm, on double substitution affects the magnetic interactions and subsequently enhances magnetic features [15]. Herein, to the best of our knowledge, the present work is the first report to describe the synthesis of cobalt ferrite/M-type strontium hexaferrite with the tartaric acid precursor strategy or sol gel auto-combustion pathway, with tartaric acid used as a fuel. The effect of the co-doping of M-type hexaferrite with La^3+^ and Sm^3+^ ions was investigated at different concentrations. The structural and magnetic properties were reorganized through X-ray powder profiling, scanning electron microscopy, transmission electron microscopy, and vibration sample magnetometry.

## 2. Experimental

**Figure 1:**
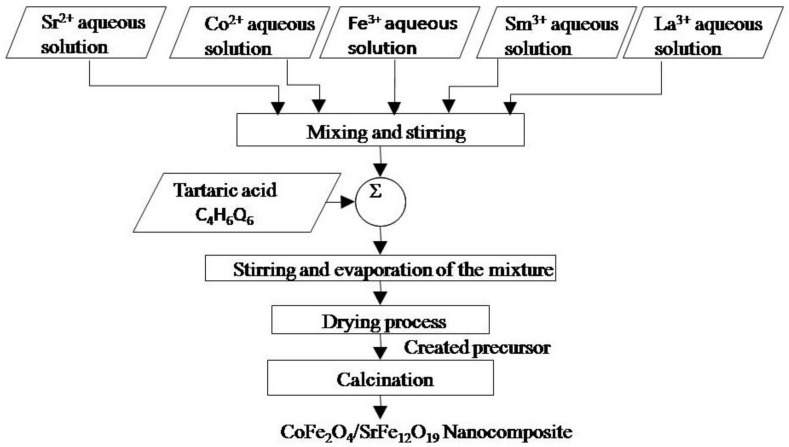
Flow-sheet diagram for synthesis of 2CoFe_2_O_4_/SrFe_12_O_19_ nanocomposite using a tartrate precursor strategy.

Spinel cobalt ferrite/strontium hexaferrite (CoFe_2_O_4_/SrFe_12−2x_Sm*_x_*La*_x_*O_19_; *x* = 0.2, 0.5, 1.0, 1.5) nanocomposites with a cobalt ferrite/strontium ratio of 2:1 were synthesized using the tartrate precursor technique. Tartaric acid was used as the chelating agent and fuel. Pure chemical grades of cobalt chloride hexahydrate (CoCl_2_.6H_2_O), strontium chloride hexahydrate (SrCl_2_.6H_2_O), ferric chloride anhydrous (FeCl_3_), lanthanum nitrate hexahydrate (La(NO_3_)_3_.6H_2_O), samarium nitrate hexahydrate (Sm(NO_3_)_3_.6H_2_O), and tartaric acid (C_4_H_6_O_6_) were added to the aqueous solution, according to the precalculated ratios of Sr^2+^, Co^2+^, Sm^3+^, La^3+^, and Fe^3+^ ions (1.0:2.0:*x*, *x*, and 16-*x*). The prepared CoFe_2_O_4_/SrFe_12−2x_Sm*_x_*La*_x_*O_19_ nanocomposites (*x* = 0.2, 0.5, 1.0, 1.5) had a content of 2:1 (Figure 1). The solutions were gently agitated on a hot plate magnetic stirrer at 90 °C until sticky gel precursors with good homogeneity were obtained. Thereafter, the formed sticky gel was dried in an oven at 110 °C overnight. Eventually, the formed precursors were annealed in a static atmosphere furnace at annealing temperatures of 1000–1200 °C for 2 h. X-ray diffraction (XRD) profiles were collected with a model Bruker AXS diffractometer, from Karlsruhe, Germany (D8-ADVANCE), with Cu Kα ((λ = 1.54056 A°) radiation, operating at 40 kV and 10 mA and used in evaluating the phase evolution in a 2θ range of 10°–70°. The average crystallite size was estimated using the well-known Debye–Scherrer equation, and according to the strongest peaks for CoFe_2_O_4_ at 2θ of 35.49°, SrFe_12_O_19_ at 2θ of 34.14°, and La_3_Co_3_O_8_ at 2θ of 32.48° [16]. The surface morphology and the average grain sizes of the created nanocomposites were examined through scanning electron microscopy (SEM) and transmission electron microscopy (TEM). A vibrating sample magnetometer (Lake Shore Cryotronics, Westerville, OH, USA) was used in studying changes in magnetic properties. Magnetic features were characterized at room temperature in a maximum applied field of 20 kOe.

## 3. Results and Discussion

### 3.1. X-ray Diffraction Characterization

**Figure 2:**
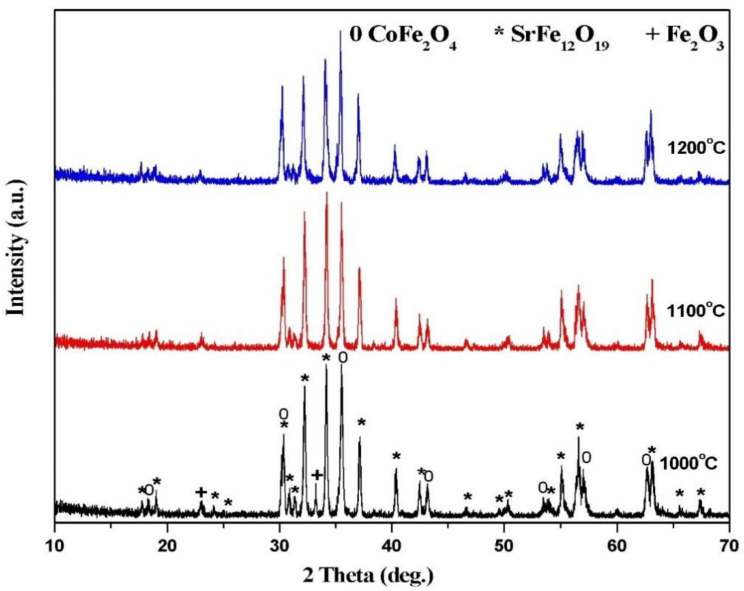
XRD patterns of 2CoFe2O4/SrFe12O19 nanocomposite tailored using a tartraric acid precursor strategy at different temperatures from 1000 to 1200 °C for 2 h.

Figure 2 shows the XRD spectra of mixed soft or hard ferrites nanocomposites without substitution annealed at various temperatures (1000–1200 °C) for 2 h. Peak profiles linked to spinel cobalt ferrite (JCPDS # 79-1744) and hexagonal SrFe_12_O_19_ (JCPDS # 84-0757) as common phases and cubic α–Fe_2_O_3_ (JCPDS # 89-0599) as an impurity secondary phase were detected at 1000 °C. Spectrum peaks corresponding to the essential diffraction planes (220), (311), (400), (422), (511), and (440) of cubic cobalt ferrite were disclosed at 2θ values of 30.32°, 35.49°_,_43.13°, 53.48°, 57.04°, and 63.05°; whereas peaks associated with the strongest diffraction planes (114), (107), (0 0 8) (110), (203), (2200), and (2011) of hexagonal SrFe_12_O_19_ were differentiated at 2θ values of 34.17°, 32.31°, 31.085°, 30.34°, 37.15°, 63.16°, and 56.8°. Otherwise, diffraction peaks at 2θ values of 33.17°, 35.66°, 54.06°, 49.46°, and 24.14° were attributed to the diffraction planes (104), (110), (116), (024), and (012), indicating the presence of an α–Fe_2_O_3_ phase. However, when the annealing temperature was increased from 1000 °C and 1200 °C within 2 h, well-defined cubic cobalt ferrite CoFe_2_O_4_ and M-type strontium hexaferrite SrFe_12_O_19_ nanocomposite was formed.

**Figure 3:**
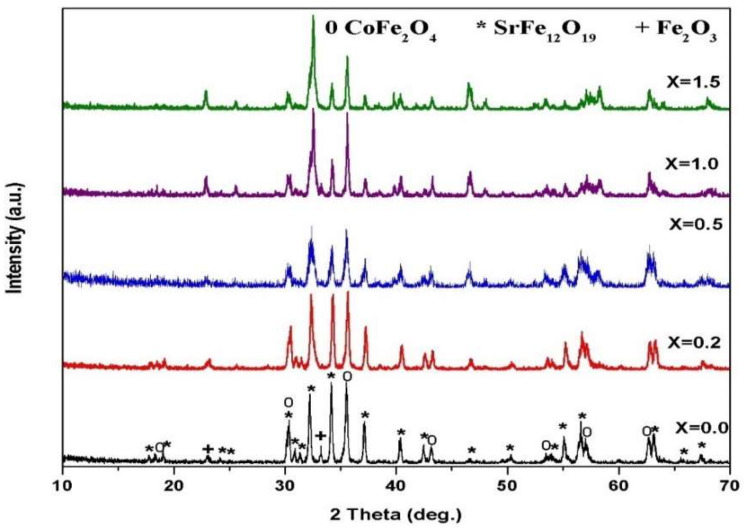
XRD patterns of 2CoFe_2_O_4_/SrFe_12−2x_Sm_x_La*_x_*O_19_ nanocomposite tailored using a tartaric acid precursor strategy at different La-Sm concentrations annealed at 1000 °C for 2 h.

**Figure 4:**
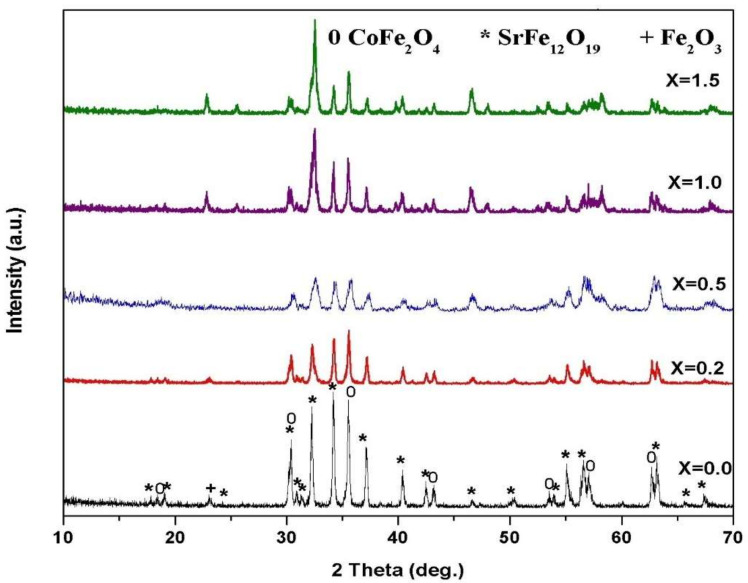
XRD patterns of 2CoFe_2_O_4_/SrFe_12−2x_Sm*_x_*La*_x_*O_19_ nanocomposite tailored using tartaric acid precursor strategy at different La-Sm concentrations annealed at 1100 °C for 2 h.

Figure 3 and Figure 4 show the SrFe_12−2x_Sm_*x*_La_*x*_O_19_ (*x* = 0.2, 0.5, 1.0, 1.5) co-doped with Sm^3+^ and La^3+^ ions at annealing temperatures of 1000–1100 °C and an annealing time of 2 h. Spinel ferrite and M-type ferrite formed in all the samples with various replacements at 1000–1100 °C. The intensities of the main peaks of strontium ferrite decreased, and the positions of the peaks slightly shifted to a low 2θ value after the concentrations of Sm^3+^ and La^3+^ ions increased.

**Figure 5:**
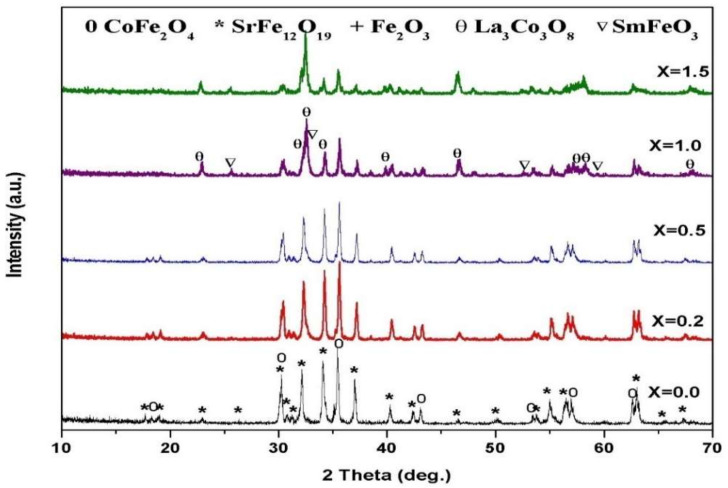
XRD patterns of CoFe_2_O_4_/SrFe_12−2x_Sm_*x*_La_*x*_O_19_ nanocomposite tailored using tartaric acid precursor strategy at different La-Sm concentrations annealed at 1200 °C for 2 h.

Figure 5 illustrates the effect of co-doping SrFe_12−2x_Sm_*x*_La_*x*_O_19_ with Sm^3+^ and La^3+^ ions on the phase evolution of the nanocomposite synthesized from the tartaric acid precursors annealed at 1200 °C for 2 h. The XRD patterns confirmed that all the samples were well-defined nanocomposites of cubic CoFe_2_O_4_ and hexagonal strontium hexaferrite with Sm^3+^-La^3+^ double substitution of ≤0.5. However, in samples with Sm^3+^–La^3+^co-doped substitution of ≥1.0, the peaks of spinel cobalt and hexagonal ferrites dramatically decreased, and new diffraction patterns were observed. Diffraction peaks for the nonmagnetic phases of La_3_Co_3_O_8_ (JCPDS # 89-1319) and SmFeO_3_ (JCPDS # 74-1474) were observed as well.

The average crystallite size of 2CoFe_2_O_4_/SrFe_12_O_19_ nanocomposite was calculated using the XRD data and the Scherrer equation. The most intense peak of each constituent phase was used in the calculation (peak at 2θ of 35.49° for the CoFe_2_O_4_ phase, peak at 2θ of 34.14° for the SrFe_12_O_19_ phase, and peak at 2θ of 35.49° for the La_3_Co_3_O_8_ phase). The calculated crystallite sizes are listed in Table 1. Notably, crystallite size increased with annealing temperature in all dominant phases (2CoFe_2_O_4_, SrFe_12_O_19_, and La_3_Co_3_O_8_). However, the crystallite size range in all the studied samples, at all constituent phases, was in a nanocrystalline range of 39.4 to 122.4 nm. In the spinel cubic CoFe_2_O_4_ phase, crystallite size decreased at Sm^3+^–La^3+^ dual displacement of up to 0.5 (at 1200 °C, from 109.7 nm at *x* of 0 to 86.1 nm at *x* of 0.5) and then increased again at a higher Sm^3+^–La^3+^ binary substitution (to 91.7 nm at *x* of 1.5; 1200 °C). Indeed, the crystallite size of hexagonal SrFe_12_O_19_ increased with Sm^3+^–La^3+^ double substitution at all *x* values. It increased from 59.5 nm at *x* of 0.0 to 122.4 nm at *x* of 1.5 (annealed at 1200 °C). The La_3_Co_3_O_8_ phase formed only at *x* of ≥1.0, and its crystallite size increase from 39.4 nm at *x* of 1.0 to 51.1 nm at *x* of 1.5. The reduction in the crystallite size with La ion content can be imputed to phase transition and the difference between ionic radii of La^3+^ (1.36 Å), Sm^3+^ (1.24 Å), and Fe^3+^ (65 Å).

### 3.2. Microstructure

**Figure 6:**
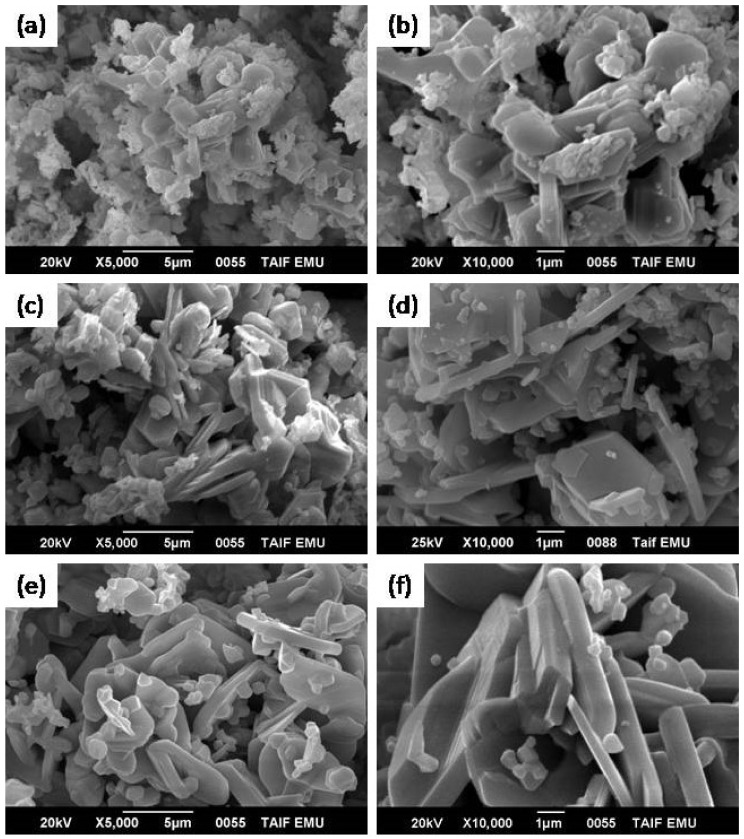
SEM micrographs of 2CoFe_2_O_4_/SrFe_12_O_19_ nanocomposite synthesized using a tartaric acid precursor strategy at different annealing temperatures, (**a**,**b**) 1000 °C, (**c**,**d**) 1100 °C, (**e**,**f**) 1200 °C for 2 h.

**Figure 7:**
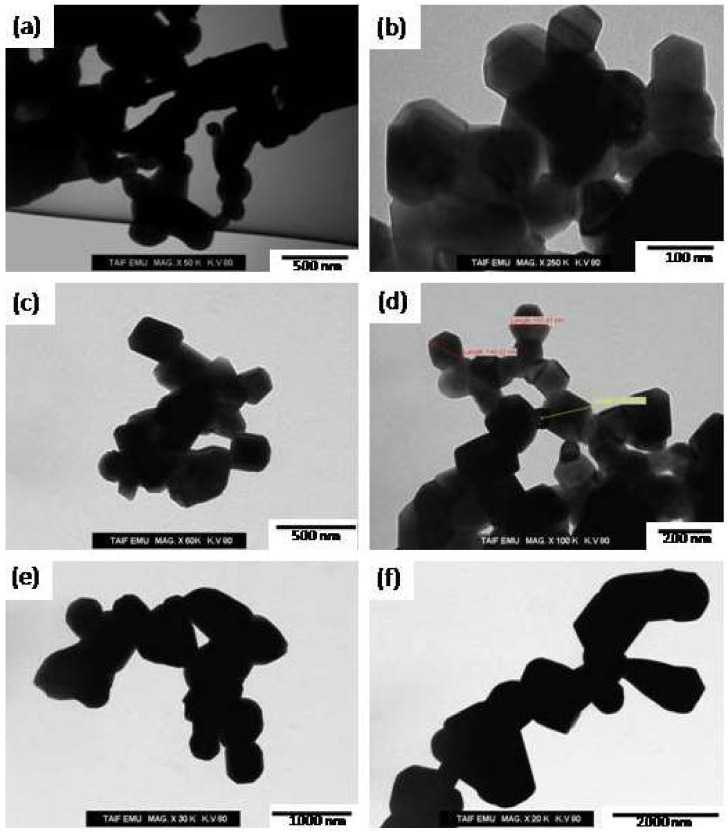
TEM micrographs of 2CoFe_2_O_4_/SrFe_12_O_19_ nanocomposite synthesized using a tartaric acid precursor strategy at different annealing temperature, (**a**,**b**) 1000 °C, (**c**,**d**) 1100 °C, (**e**,**f**) 1200 °C for 2 h.

**Figure 8:**
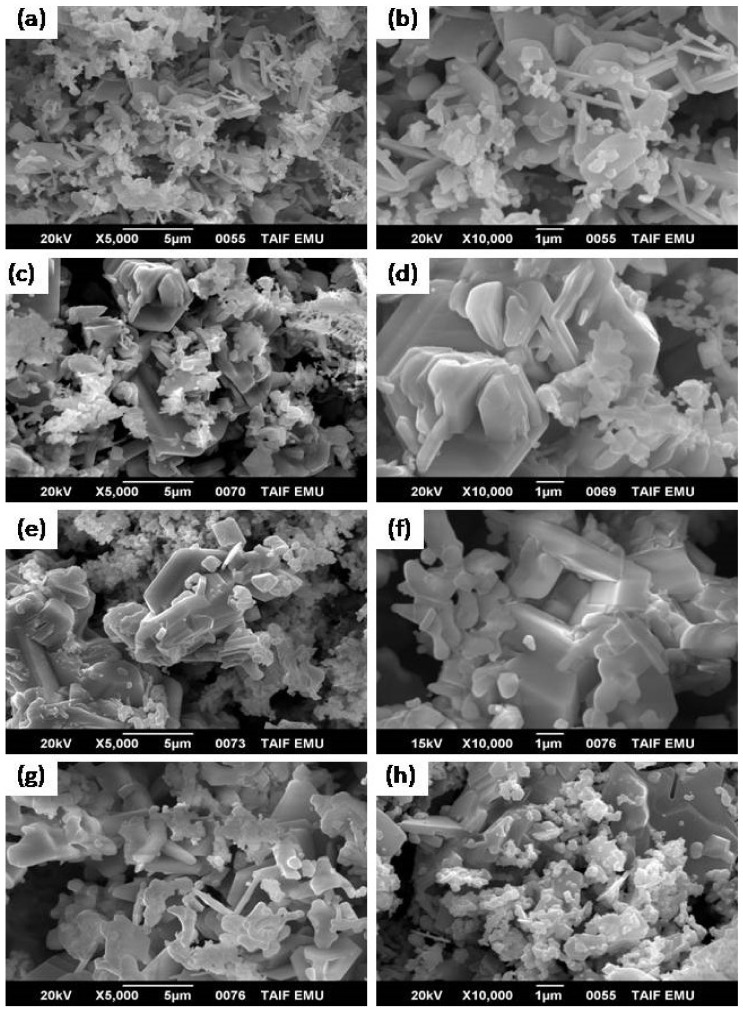
SEM micrographs of 2CoFe_2_O_4_/ SrFe_12-x_La_0.5x_Sm_0.5x_O_19_ nanocomposite tailored using a tartaric acid precursor strategy at 1100 °C, (**a**,**b**) 2CoFe_2_O_4_/ SrFe_11.6_La_0.2_Sm_0.2_O_19_ (**c**,**b**) 2CoFe_2_O_4_/ SrFe_11_La_0.5_Sm_0.5_O_19_ (**e**,**f**) 2CoFe_2_O_4_/ SrFe_10_La_1.0_Sm_1.0_O_19_ (**g**,**h**) 2CoFe_2_O_4_/SrFe_9_La_1.5_Sm_1.5_O_19_ for 2 h.

Figure 6 shows SEM images of the produced spinel ferrite or M-type hexaferrite at different annealing temperatures. A fine spherical structure with random grain orientation and hexagonal platelet-like structures related to strontium M-type ferrite was observed in the samples annealed at 1000 °C (Figure 6a,b). This temperature was insufficient for the formation of crystallite phases of soft or hard nanocomposites. As the temperature was increased to 1100 °C (Figure 6c,d), the grains started to grow again, and the SEM micrographs showed a clear aggregation of crystallite hexagonal platelet particles (SrFe_12_O_19_) with sharp planes of crystals coated by small spherical or cubic grains (CoFe_2_O_4_). The same structures were observed in the samples annealed at 1200 °C. In addition, multiple layers of hexagonal platelet-like structures coated by cubic grains (CoFe_2_O_4_) and crystals with uniform coarse structures were observed. The accumulation of spherical (or cubic) grains on the surface of the hexagonal platelet-like structure in the SrFe_12_O_19_ hard ferrite indicated magneto-dipole interactions among the particles of the soft and hard ferrites [17]. However, the grain size of the hard ferrite phase was larger than that of the soft ferrite phase. Figure 7 shows the TEM micrographs of the synthesized nanocomposites at different annealing temperatures without metal substitution. Large hexagonal particles with small particles were observed in the nanocomposites. The TEM micrographs showed that the sizes of the hard and soft ferrite nanocomposites were several tens of nanometers at an annealing temperature of 1000 °C (Figure 7a,b). The size range of the synthesized nanocomposite increased to several hundreds of nanometers at an annealing temperature of 1200 °C (Figure 7e,f). The effect of Sm^3+^ and La^3+^ ion substitution on the produced 2CoFe_2_O_4_/SrFe_12−2x_Sm*_x_*La*_x_*O_19_ (*x* = 0.2, 0.5, 1.0, 1.5) annealed at 1100 °C is evident in the SEM micrograph presented in Figure 8. The composition of SrFe_12_O_19_ indicated a hexagonal platelet-like structure with accumulated spherical grains and the pseudo-cubic shapes of different components on the surface of the hexagonal plate. At an *x* of 0.2, the morphology showed a platelet-like structure of hexagonal strontium hexaferrite, accumulated spherical grains, few rod-like structures, and the pseudo-cubic structure of cobalt ferrite specimen (Figure 8a,b). The number and size of the platelet-like structures of hexagonal ferrite and rod-like structures of soft ferrite increased at an La^3+^–Sm^3+^ ion ratio of 0.5 (Figure 8c,d). Furthermore, at an *x* of ≥1.5 (Figure 8e–h), some plate-like hexagonal strontium hexaferrite (the distribution of spherical or pseudo-cubic grains structures corresponding to soft ferrite), La_3_Co_3_O_8_, and SmFeO_3_ were observed. To clarify the distribution of each element (Sr, Co, Fe, and O) in the structure, EDX spot analysis of the 2CoFe_2_O_4_/SrFe_12_O_19_ nanocomposite powders (without substitution) annealed at 1200 °C was conducted. The range of analysis for all reported phases is provided in Table 2. The concentration range of cobalt in the small cubic structure phase (9.13–14.61 atom %) was higher than that in the plate structure (2.82–3.16 atom %). Moreover, the concentration range of strontium in the small pseudo-cubic structure phase (0.40–0.88 atom %) was lower than that in the plate structure (3.55–3.85 atom %). These results indicated that the spherical structure phase was spinel CoFe_2_O_4_, whereas the plate structure phase was hexagonal ferrite (SrFe_12_O_19_). However, the concentration range of iron in the plate structure (35.69–65.85 atom %) was higher than the spherical structure phase (27.72–41.70 atom %), whereas the concentration range of oxygen in the plate structure (24.14–58.94 atom %) was lower than that in the spherical structure phase (43.29–62.72 atom %). The concentration ranges of iron and oxygen indicated that the spherical structure phase was spinel CoFe_2_O_4_ and the plate structure phase was hexagonal ferrite (SrFe_12_O_19_).

### 3.3. Magnetic Properties

**Figure 9:**
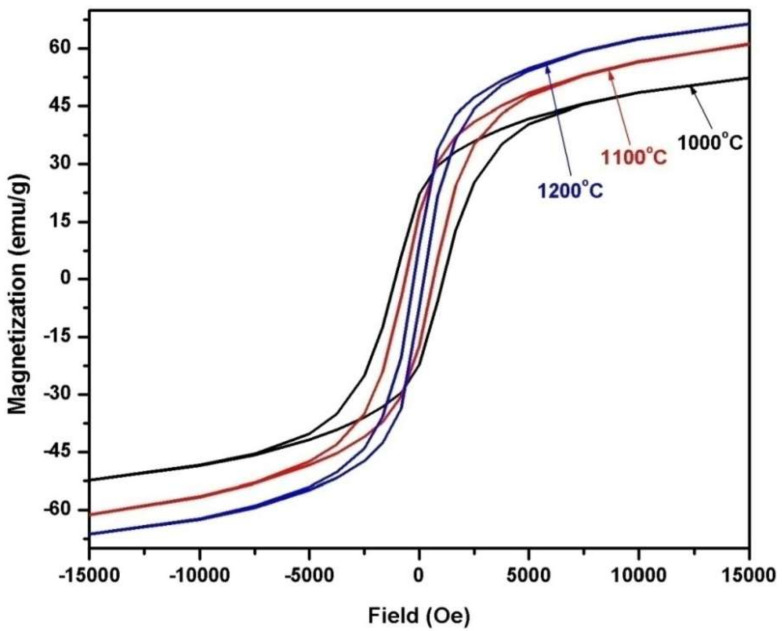
*M-H* loops of 2CoFe_2_O_4_/SrFe_12_O_19_ nanocomposite powders tailored using a tartaric acid precursor strategy with different annealing temperatures from 1000 to 1200 °C for 2 h.

**Figure 10:**
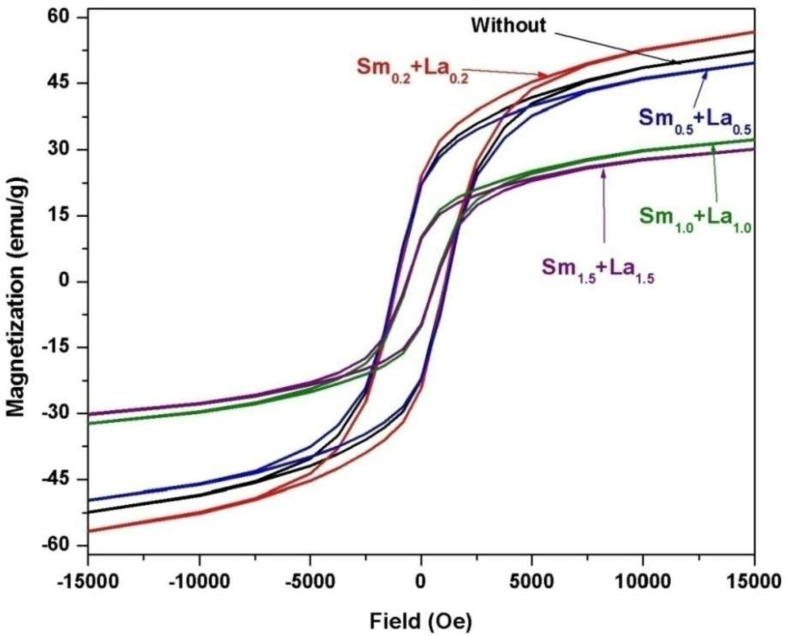
*M-H* loops of 2CoFe_2_O_4_/SrFe_12−2x_Sm*_x_*La*_x_*O_19_ nanocomposite powders tailored using tartaric acid precursor strategy with different La-Sm ratios at annealing temperature 1000 °C for 2 h.

**Figure 11:**
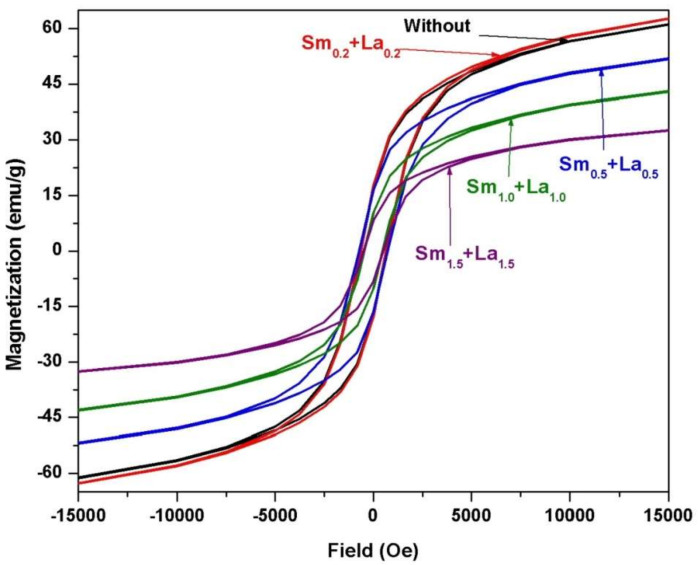
*M-H* loops of 2CoFe_2_O_4_/SrFe_12−2x_Sm*_x_*La*_x_*O_19_ nanocomposite powders tailored using tartaric acid precursor strategy with different La-Sm ratios at annealing temperature1100 °C for 2 h.

**Figure 12:**
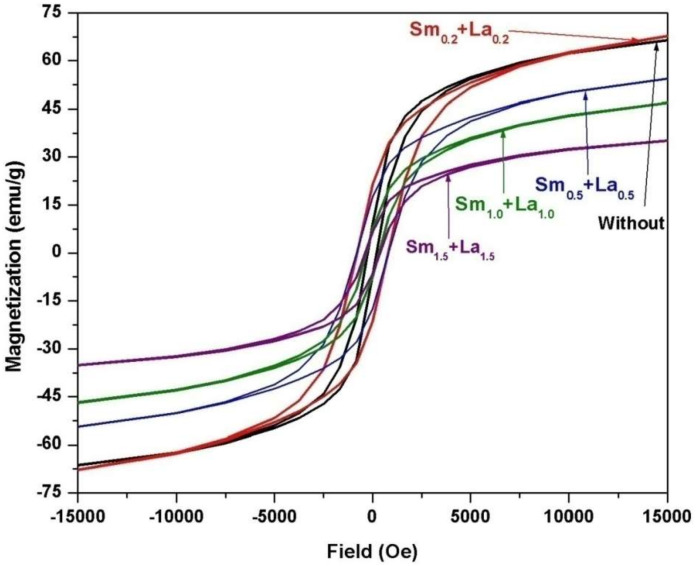
*M-H* loops of 2CoFe_2_O_4_/SrFe_12−2x_Sm*_x_*La*_x_*O_19_ nanocomposite powders tailored using tartaric acid precursor strategy with different La-Sm ratios at annealing temperature1200 °C for 2 h.

**Figure 13:**
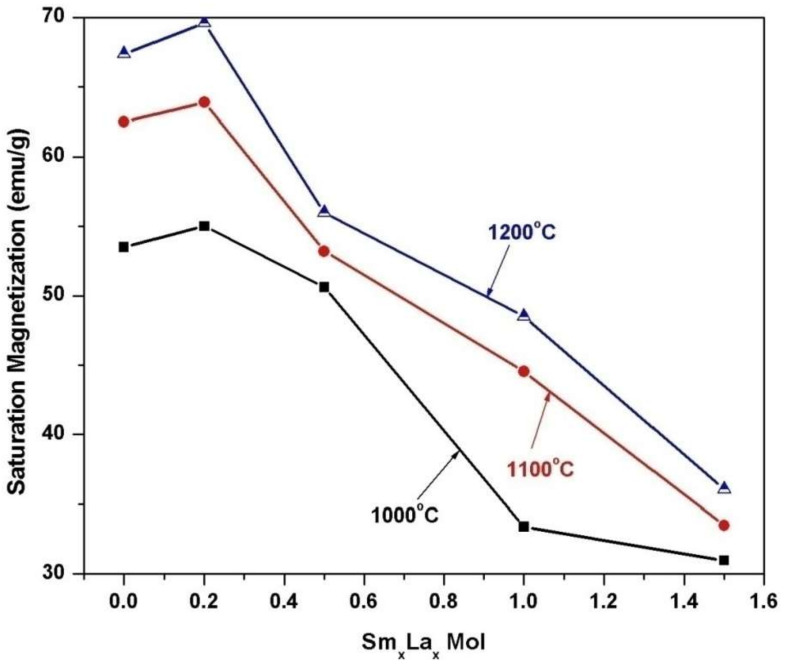
Effect of Sm^3+^-La^3+^ substitution and annealing temperature on the saturation magnetization (*Ms*) of 2CoFe_2_O_4_/SrFe_12−2x_Sm*_x_*La*_x_*O_19_ nanocomposite powders tailored using tartaric acid precursor strategy.

**Figure 14:**
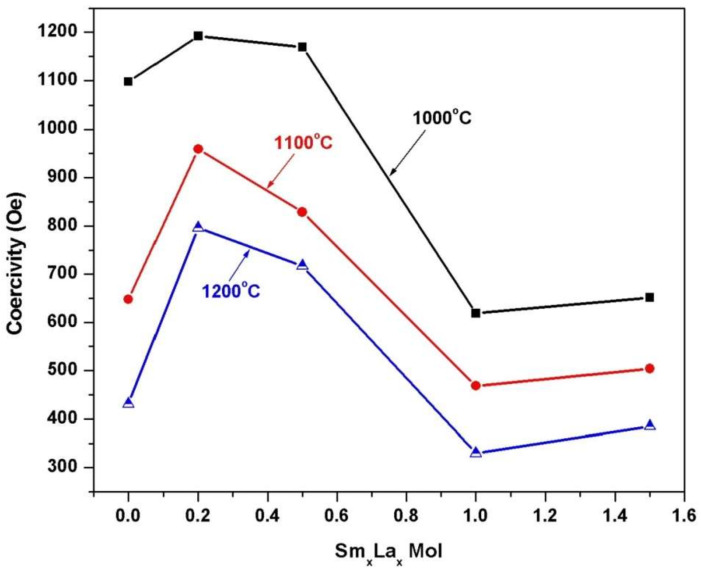
Effect of Sm^3+^-La^3+^ substitution and annealing temperature on the coercivity (*Hc*) of 2CoFe_2_O_4_/SrFe_12−2x_Sm_x_La*_x_*O_19_ nanocomposite powders tailored using a tartaric acid precursor strategy.

Figure 9 includes the field dependence of the magnetization of 2CoFe_2_O_4_/SrFe_12_O_19_ samples synthesized at different temperatures (1000–1200 °C) for 2 h. The measurements were performed at room temperature, and the maximum applied field was 20 kOe. The magnetic properties were intermediate between the two phases. The formed nanocomposites showed single-phase magnetic characteristics, indicating that the magnetic hard and soft phases were exchange-coupled [18]. The saturation magnetization *Ms* value increased with annealing temperature. The *M*_s_ value gradually increased from 53.5 emu/g at 1000 °C to 67.4 emu/g at 1200 °C. Variations in saturation magnetization were observed as the crystallite size increased in the two formed magnetic phases. Furthermore, the saturation magnetization increased with the decreasing ratio of nonmagnetic species of α–Fe_2_O_3_. However, the coercive force *H*_c_ of the composite decreased with increasing annealing temperature. As a result, the *α*-Fe_2_O_3_ presented showed a high intrinsic coercive force at an annealing temperature of 1000 °C. Furthermore, changes in this value may be attributed to increase in the soft cobalt ferrite phase at increased annealing temperature [19]. Coercivity decreased with crystallite size and with lattice imperfections, voids, and porosity, attributed to the multidomain formation and the facile movement of domain walls [4]. Figure 10, Figure 11 and Figure 12 show the magnetic hysteresis loops (*M–H* curves) of the 2CoFe_2_O_4_/SrFe_12−2x_Sm_*x*_La_*x*_O_19_ nanocomposite powder synthesized at different temperatures (1000–1200 °C). Table 3 lists the estimated values of magnetic parameters, namely, saturation magnetization (*M*_s_), residual magnetization (*M*_r_), coercivity field (*H*_c_), and squareness ratio (*Mr*/*Ms*). The impacts of La^3+^ and Sm^3+^ content on the saturation magnetization and coercive force of the formed nanocomposites are illustrated in Figure 13 and Figure 14. Remarkably, the saturation magnetization and coercive force of 2CoFe_2_O_4_/SrFe_11.6_Sm_0.2_La_0.2_O_19_ increased with annealing temperature. The lanthanide element is usually established in the octahedral site (12k, 2a, 4f_2_), because of volume impact. Moreover, La^3+^ and Sm^3+^ ions preferentially occupy the 4f_2_ site. Fe^3+^ ions occupy five sites in the strontium hexaferrite structures (three octahedral sites with spin up direction (12k, 2a) and spin down direction (4f_2_), one tetragonal site with spin down direction (4f_1_), and one trigonal bipyramidal site with spin up direction (2b). At the 4f_2_ site, Fe with spin down moment is replaced by rare earth elements, indicating that it has a lower magnetic moment (La^+3^ and Sm^+3^ ions have 1.5 µ_B,_ unpaired electron [4f^5^] and 0 µ_B_ [4f^0^], respectively) than Fe^+3^ ion (5 µ_B_). The overall magnetic moment increased, and, thus, the total magnetization of the nanocomposite increased. However, as the substitution ratios increased from 0.5 to 1.5, La^3+^ and Sm^3+^ ions occupied the spin-up sites (2a, 2b, and 12 k), or La^3+^ ions were substituted by the Fe^3+^ ions in the spin up sites, the net magnetic moment and magnetization decreased. This result may be attributed to the formation of the impure secondary phases of the nonmagnetic phases of La_3_Co_3_O_8_ and SmFeO_3_, as indicated by the XRD profile data_._ Furthermore, high concentrations of La^+3^ and Sm^+3^ ions steadily altered the magnetization of the Fe^+3^ ion, from a collinear spin to a noncollinear spin. Additionally, substitution by La^+3^ and Sm^+3^ decreased the strength of the super-exchange interaction between Fe^+3^-O-Fe^+3^, leading to the non-collinear or spin-canting arrangement of magnetic moments [20,21]. The squareness ratio *Mr/Ms* ranged from 1.5 to 0.46, confirming that the formed nanocomposites were in multimagnetic domains [16].

## 4. Conclusions

The results are summarized as the follows:

- Soft or hard ferrite nanocomposite, based on cobalt ferrite CoFe_2_O_4_/strontium hexaferrite SrFe_12_O_19_ in a ratio of 2:1, was successfully synthesized for the first time with tartaric acid precursor, at annealing temperatures of 1100–1200 °C and an annealing time of 2 h.
- The simultaneous insertion La^3+^ and Sm^3+^ ions into CoFe_2_O_4_/SrFe_12−2x_Sm*_x_*La*_x_*O_19_ nanocomposites (*x* = 0.2, 0.5, 1.0, 1.5) indicated that the two phases did not deteriorate until *x* > 1.5 because of the impurities in the La_3_Co_3_O_8_ and SmFeO_3_ phases produced.
- The microstructures of the two formed magnetic phases were affected by the ratio between the CoFe_2_O_4_ and SrFe_12_O_19_ phases and the La3+ and Sm3+ ion substitution ratio. The morphology of the pure nanocomposites displayed hexagonal platelet-like structures with pseudo-cubic shapes.
- EDX spot analysis revealed that Fe, Sr, O, and Co elements were distributed in the cubic and hexagonal plate-like structures, and large concentrations of Fe and Sr were found in the plate- like structures.
- The occupation of La-Sm co-doped ions in the lattice sites of the spin down magnetic moments 4f_2_ led to an increase in net magnetic moments in the magnetic structure of M-type hexaferrite, which in turn increased the *Ms* value. However, when the substitution ratios increased from 0.5 to 1.5, La^3+^ and Sm^3+^ occupied the spin up sites (2a, 2b, and 12 k), or when the La^3+^ and Sm^3+^ ions were substituted by the Fe^3+^ ions in the spin up sites, the net magnetic moment and magnetization decreased.
- A high saturation magnetization value (*Ms* = 69.6 emu/g) and high coercive force value (*Hc* = 1192.0Oe) were obtained at annealing temperatures 1200 °C and 1000 °C, respectively, at a La^3+^–Sm^3+^ co-doped ratio of 0.2.
- These nanocomposites are potential materials for microwave absorber devices and hyper-frequency applications, such as multilayer chip inductors and LC filters.

## Figures and Tables

**Table 1:** Crystallite size variation for the formed phases with Sm^3+^-La^3+^ double substituted CoFe_2_O_4_/SrFe_12−2x_Sm*_x_*La*_x_*O_19_ annealed at 1000, 1100 and 1200 °C elaborated using tartaric acid precursor approach (CoFe_2_O_4_ at 2θ = 35.49, SrFe_12_O_19_ at 2θ = 34.14, La_3_Co_3_O_8_ at 2θ = 32.48).

Composition	Temperature, °C	Formed Phase	C. Size, nm
2CoFe_2_O_4_/SrFe_12_O_19_	1000	CoFe_2_O_4_	79.6
		SrFe_12_O_19_	42.2
	1100	CoFe_2_O_4_	94.6
		SrFe_12_O_19_	51.8
	1200	CoFe_2_O_4_	109.7
		SrFe_12_O_19_	59.5
2CoFe_2_O_4_/SrFe_11.6_Sm_0.2_La_0.2_O_19_	1000	CoFe_2_O_4_	75.1
		SrFe_12_O_19_	60.9
	1100	CoFe_2_O_4_	83.2
		SrFe_12_O_19_	89.4
	1200	CoFe_2_O_4_	91.4
		SrFe_12_O_19_	101.1
2CoFe_2_O_4_/SrFe_11_Sm_0.5_La_0.25_O_19_	1000	CoFe_2_O_4_	72.6
		SrFe_12_O_19_	76.2
	1100	CoFe_2_O_4_	79.5
		SrFe_12_O_19_	86.6
	1200	CoFe_2_O_4_	86.1
		SrFe_12_O_19_	105.4
2CoFe_2_O_4_/SrFe_10_Sm_1.0_La_1.0_O_19_	1000	CoFe_2_O_4_	70.1
		SrFe_12_O_19_	80.1
	1100	CoFe_2_O_4_	74.9
		SrFe_12_O_19_	96.8
	1200	CoFe_2_O_4_	88.2
		SrFe_12_O_19_	113.7
		La_3_Co_3_O_8_	39.4
2CoFe_2_O_4_/SrFe_9_Sm_1.5_La_1.5_O_19_	1000	CoFe_2_O_4_	76.0
		SrFe_12_O_19_	87.9
	1100	CoFe_2_O_4_	84.1
		SrFe_12_O_19_	107.3
	1200	CoFe_2_O_4_	91.7
		SrFe_12_O_19_	122.4
		La_3_Co_3_O_8_	51.1

**Table 2:** Spot Analysis Range of Constituent Elements in the 2CoFe_2_O_4_/SrFe_12_O_19_, wt% nanocomposite powders (without substitution) annealed at 1200 °C fabricated using a tartraic acid precursor approach.

Element	Co		Sr		Fe		O	
	Mass%	Atom%	Mass%	Atom%	Mass%	Atom%	Mass%	Atom%
Plate-Like structure	4.81–5.54	2.82–3.16	10.15–10.49	3.55–3.85	57.73–72.94	35.69–65.85	7.52–27.31	24.14–58.94
Small cubic crystals	17.08–21.87	9.13–14.61	1.41–3.43	0.40–0.88	49.16–59.13	27.72–41.70	17.59–31.86	43.29–62.72

**Table 3:** Effects of Sm^3+^-La^3+^ substitution and annealing temperature on the magnetic properties of 2CoFe_2_O_4_/SrFe_12−2x_Sm_*x*_La_*x*_O_19_ elaborated at different La-Sm concentrations and annealing temperatures tailored using a tartaric acid precursor approach.

Composition	Temperature, (°C)	Magnetic Properties			
		Saturation Magnetization *M_s_*, (emu/g)	Retentivity *M_r_*, (emu/g)	Coercivity *H_c_*, (Oe)	*M_r_*/*M_s_*
2CoFe_2_O_4_/SrFe_12_O_27_	1000	53.5	21.3	1097.6	0.3981
	1100	62.5	17.4	648.0	0.2784
	1200	67.4	14.1	431.13	0.2092
2CoFe_2_O_4_/ SrFe_11.6_Sm_0.2_La_0.2_O_19_	1000	55.0	23.8	1192.0	0.4327
	1100	63.9	22.4	959.0	0.3505
	1200	69.6	21.5	795.9	0.3089
2CoFe_2_O_4_/ SrFe_11_Sm_0.5_La_0.5_O_19_	1000	50.6	23.4	1169.2	0.4625
	1100	53.2	16.5	828.5	0.3102
	1200	56.0	16.9	716.7	0.3018
2CoFe_2_O_4_/ SrFe_10_Sm_1.0_La_1.0_O_19_	1000	33.4	10.1	618.4	0.3024
	1100	44.5	10.3	468.9	0.2315
	1200	48.5	7.5	329.5	0.1546
2CoFe_2_O_4_/ SrFe_9_Sm_1.5_La_1.5_O_19_	1000	31.0	9.7	651.7	0.3129
	1100	33.5	8.3	503.7	0.2478
	1200	36.1	6.6	384.4	0.1828

## Data Availability

Not applicable.
